# Thoracic, Lumbar, and Sacral Pedicle Screw Placement Using Stryker-Ziehm Virtual Screw Technology and Navigated Stryker Cordless Driver 3: Technical Note

**DOI:** 10.3390/jcm7040084

**Published:** 2018-04-17

**Authors:** Praveen Satarasinghe, Kojo D. Hamilton, Michael J. Tarver, Robert J. Buchanan, Michael T. Koltz

**Affiliations:** 1 Division of Neurosurgery, Department of Surgery and Perioperative Care, Dell Medical School, University of Texas at Austin, Austin, TX 78712, USA; praveen.satarasinghe@utexas.edu (P.S.); micheal.j.tarver@utexas.edu (M.J.T.); robert.buchanan@utexas.edu (R.J.B.); 2 Department of Neurosurgery, University of Pittsburgh, Pittsburgh, PA 15213, USA; hamiltondk@upmc.edu; 3 Department of Neurosurgery, Seton Brain and Spine Institute, Austin, TX 78712, USA

**Keywords:** pedicle screw, virtual technique, neuronavigation, spine, surgery, three-dimensional

## Abstract

Object. Utilization of pedicle screws (PS) for spine stabilization is common in spinal surgery. With reliance on visual inspection of anatomical landmarks prior to screw placement, the free-hand technique requires a high level of surgeon skill and precision. Three-dimensional (3D), computer-assisted virtual neuronavigation improves the precision of PS placement and minimization steps. Methods. Twenty-three patients with degenerative, traumatic, or neoplastic pathologies received treatment via a novel three-step PS technique that utilizes a navigated power driver in combination with virtual screw technology. (1) Following visualization of neuroanatomy using intraoperative CT, a navigated 3-mm match stick drill bit was inserted at an anatomical entry point with a screen projection showing a virtual screw. (2) A Navigated Stryker Cordless Driver with an appropriate tap was used to access the vertebral body through a pedicle with a screen projection again showing a virtual screw. (3) A Navigated Stryker Cordless Driver with an actual screw was used with a screen projection showing the same virtual screw. One hundred and forty-four consecutive screws were inserted using this three-step, navigated driver, virtual screw technique. Results. Only 1 screw needed intraoperative revision after insertion using the three-step, navigated driver, virtual PS technique. This amounts to a 0.69% revision rate. One hundred percent of patients had intraoperative CT reconstructed images taken to confirm hardware placement. Conclusions. Pedicle screw placement utilizing the Stryker-Ziehm neuronavigation virtual screw technology with a three step, navigated power drill technique is safe and effective.

## 1. Introduction

The development of minimally invasive techniques that replace traditional open spine surgeries has decreased the incidence of complications, approach-related morbidity and mortality, bleeding, infection, postoperative pain, and hospital stay [1,2]. One of these techniques is image-guided surgeries, which have demonstrated their ability to improve patient outcomes relative to free hand techniques which rely only upon physical landmarks [3,4]. Well positioned pedicle screw placement is essential due to the precarious anatomical area in which the screws are placed in proximity to the spinal cord, spinal roots, and systemic and neuro-vasculature. With the classic free-hand technique for screw positioning, there is a greater likelihood for disastrous complications with pedicle screws (PS) placement, as this technique relies on physician experience and skill rather than a digital virtual template [5,6,7]. Image-guided placement of pedicle screws appeases this problem during spinal surgeries by offering increased visualization of a pedicle’s trajectory [8]. However, image-guided PS placement still has a few shortcomings. Imaging results in radiation exposure, increased time expenditure, and possible workflow interruption [3]. Also, patient movement in relation to the reference array may cause the system to become inaccurate.

Despite the shortcomings, image-guided PS placement has increased in popularity recently among spine surgeons due to the decrease in breach rate and improvements in PS placement accuracy [9]. From analysis of the current literature, it is apparent that pedicles of vertebrae are difficult to work with, as they can have altered morphology that makes them difficult to cannulate [9]. A systematic review of the accuracy of PS placement has documented the misplacement rates for lumbar and thoracic PS placement using the free-hand technique to be as high as 41 and 55 percent, respectively [5]. In contrast, in the same systematic review, misplacement rates for PS placement using computer-assisted neuronavigation have been estimated to be significantly lower, ranging from 89 to 100 percent [5].

The authors of this paper demonstrate a three-step virtual PS technique for thoracic, lumbar, and sacral spinal levels that minimizes misplacement rates and complications.

## 2. Methods

Patients were evaluated and treated at a single institution by the senior author. Pathology which the senior author felt needed instrumentation for adequate treatment included degenerative, traumatic, and neoplastic cases. A database was generated and outcomes analyzed according to the Health Insurance Portability and Accountability Act (HIPAA) compliance. Each selected patient had at least three months of outcome data for analysis at the time of manuscript preparation.

**Figure 1:**
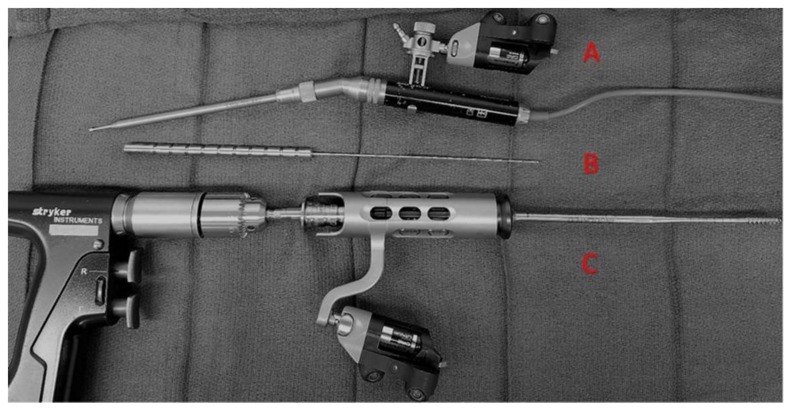
Instruments needed for safe placement of thoracic, lumbar, and sacral pedicle screws using the virtual screw technique. (**A**) Stryker electric drill with navigation tracker interface and 3-mm match stick drill bit. (**B**) Pedicle feeler. (**C**) Stryker Instruments Cordless Driver 3 with navigated tip interface coupling ring, tracker interface, handle interface, and tap.

All procedures were done on a Jackson table with Stryker NAV3i Surgical Navigation Platform (Stryker Navigation, Kalamazoo, MI, USA) linked to the Ziehm Vision RFD 3D (Ziehm Imaging GmbH, Nuremberg, Germany). If upper thoracic screws were planned, the Mayfield head clamp was used and attached to the Jackson table with Mayfield tower system. The highest instrumented level was thoracic level 1 (T1). The Stryker electric drill and Stryker Instruments Cordless Driver 3 are registered according to Stryker protocol so that they may be navigated during the case. This assembly can be seen on Figure 1. Neuro-monitoring was used for each case. Once the appropriate patient positioning was achieved, the Ziehm Vision RFD 3D was used to localize the operative levels and skin marked accordingly. A traditional open approach was utilized to allow for adequate visualization of anatomic structures to help confirm accuracy of hardware placement, as this is a new technique and system. Although an open approach was used while developing this new technique, it is not required for successful placement of PS via the three-step virtual PS method developed by the senior author. Soft tissue is retracted with the Stryker LITe Midline Retractor (TEDAN Surgical, Sugarland, TX, 77478) to limit artifact for the intraoperative CT. The senior author feels that this was an important safety checkpoint for the patients.

Once an adequate exposure was achieved, a spinous process clamp was securely attached to the most caudal level. The Ziehm Vision RFD 3D was brought into the field after protecting the sterile field with a 360 degree sterile drape that cocooned the patient, bed, and instruments. AP and lateral X-rays are taken to make sure the levels to be instrumented are adequately visualized. An intraoperative CT is then done and images sent to the Stryker NAV3i Surgical Navigation workstation. Confirmation of correct operative levels and adequate image quality is done on the workstation. The workstation also allows for the measurement of an appropriate pedicle screw. 

Once the workstation is ready for navigation, the navigated Stryker electric drill is tested for accuracy on the exposed boney anatomy. Traditional pedicle screw entry points at the junction of the transverse process and lateral facet near the mammillary process were used to verify neuronavigation and confirm visualization of exposed anatomy. If any deviation is seen, the navigated Stryker electric drill is re-registered, and, if needed, another intraoperative CT may be performed.

**Figure 2:**
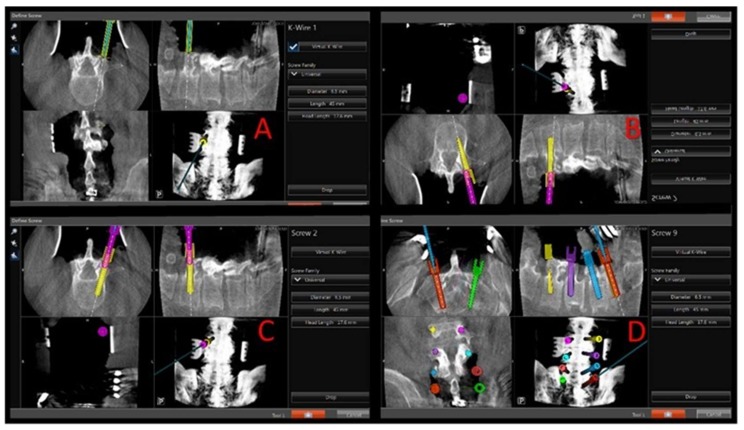
Intraoperative views of navigation screen showing steps of virtual screw placement. (**A**) Navigated Stryker drill with 3 mm match stick drill bit at anatomical entry point with screen projection showing a 6.5 × 50 mm virtual screw. (**B**) Navigated Stryker Driver with 5.5 tap accessing the vertebral body through the pedicle with screen projection again showing 6.5 × 50 screw. (**C**) Navigated Stryker driver with 6.5 × 50 screw with screen projection showing the same 6.5 × 50 virtual screw. The instruments used at each step are color-coded to avoid confusion on the navigation monitor while advancing into the pedicle and vertebral body (match stick drill bit in “A”, green. Tap in “B”, yellow. Screw in “C”, pink. (**D**) Screen shot showing final screw placement before intra-operative CT done to confirm placement.

Once accuracy is verified, the navigated Stryker electric drill with 3-mm matchstick drill bit is used to make an entry hole into the proximal 1/2 of the pedicle at the appropriate angles given by the navigation system. The Stryker navigation system is calibrated such that the matchstick drill bit projects onto the navigation screen at the desired screw diameter and length (see Figure 2). The appropriate length and diameter of the screw was selected by visualizing virtual screw projection on the Stryker workstation using intraoperative imaging. The pedicle feeler is then used to confirm access into the pedicle. The navigated Stryker Instruments Cordless Driver 3 is then brought into the file and tested for accuracy. There should be an exact match for the hole made by the Stryker electric drill. If there is not an exact match, the system needs to be checked again as described above. The navigated Stryker Cordless Driver 3 with appropriate tap (see Figure 1) is then used to access the vertebral body. The powered taps were manufactured by Stryker specifically for the senior author. Again, the tap bit projects onto the navigation screen at the desired screw diameter and length (see Figure 2). This technique helps assure that a breach will not occur when the actual screw is inserted. The pedicle feeler is again used to check for a breach after the tap is removed.

Finally, the appropriate screw is placed onto the navigated Stryker Instruments Cordless Driver 3 and tested for accuracy as before. If there is not a perfect match on the navigation screen and previously drilled hole, the system is once again tested as before. The pedicle screw is then inserted using the navigated Stryker Instruments Cordless Driver 3. Each screw is then stimulated. These steps are completed at each level. Once all levels are instrumented, a second intraoperative CT is completed to confirm adequate placement of hardware. Neuro-monitoring and post-screw placement imaging with use of intraoperative X-ray and CT scan was used to determine the acceptability of screw placement.

Twenty-three patients were selected for analysis based on appropriateness of virtual screw technique for their presenting pathology. Degenerative, traumatic, and neoplastic cases were included in the subject pool (Table 1). The number of screws needing realignment or repositioning after intraoperative CT and PS placement were compared to the total number of PS inserted into patients using the virtual technique to calculate percent of screws properly placed and percent of screws misplaced.

## 3. Results

Twenty-three consecutive patients, aged 19 to 65, were retrospectively reviewed. A total of 144 pedicle screws were inserted using the described technique. Thirty-eight screws were inserted into the thoracic spine, 90 screws were inserted into the lumbar spine, and 16 screws were inserted into the sacral spine. After insertion of screws into the 23 patients selected for analysis using the three-step virtual PS placement technique, only 1 out of the 144 inserted screws needed revision (0.69%). The screw needed to be revised due to a lateral breach at lumbar level 2 (L2). Neuro-monitoring stimulation of the screw at less than 10 millihertz (mHz) helped to confirm a cortical breach. The breach was in agreement with neuro-monitoring. This revision was done at the time of the initial surgery after routine, post-hardware placement, intraoperative CT. The single screw that was not initially successfully inserted was replaced with neuronavigation into the appropriate position, corrected using the same technique described above.

There was a 0% morbidity and mortality rate with this technique.

## 4. Discussion

Pedicle screws were first introduced into medical procedures in the 1950s and 1960s [10,11]. Since then, the utilization of pedicle screws in spine surgery has become very common. PS use during spinal surgeries has improved fusion rates and rigidity while minimizing complications associated with previous rod and hook systems [12,13,14]. Initially, pedicle screws were placed mostly in the lumbar spine, as lower spinal levels and the cauda equina are not as susceptible to neural damage [15]. Now, pedicle screw instrumentation is almost exclusively used when securing fusion constructs in the thoracic, lumbar, or sacral spine [9].

Currently, techniques can be broken down into two major categories: freehand and assisted approaches. Free-hand approach is the most common and involves the fixation of a pedicle screw without an imaging aid or with fluoroscopy, whereas assisted techniques rely on neuronavigation technology to visualize anatomical landmarks [9]. Many studies have analyzed the accuracy of both free-hand and assisted techniques.

The novelty of our assisted technique, described in the methods section, advances PS placement as compared to traditional assisted approaches through a technique that minimizes steps and equipment needed using innovative instrumentation, while also producing excellent patient outcomes. This is accomplished by (1) use of a navigated power drill eliminating the need for a manual pedicle finder, manual tap, and manual screw placement; (2) utilization of Stryker-manufactured power taps and driver attachment specifically fabricated for the senior author (Figure 1); and (3) virtual screw technology that projects final screw size on the workstation, while real-time work within the pedicle is with smaller drills and taps. Points one and two are important because they reduce the force needed to access the pedicle. The navigated Stryker Instruments Cordless Driver 3 with Stryker taps allows easy access to the vertebral body through the pedicle. As such, the spine does not move and reduces a potential source of inaccuracy in spinal navigation. Point three is important because even when navigating instruments that are smaller in size than the final screw that is to be placed, such as the 3-mm match stick drill-bit, virtual screw size and consequent fit of the actual screw within patient anatomy are able to be visualized with great accuracy, minimizing the chance of a breach.

The novelty of this technique is a combination of using a power driver with virtual screw technology. The visualization approach comes from the initial utilization of only a 3-mm tip and the screen projection of a real-size virtual screw. Using a small 3-mm tip prevents complications and potentially harmful entry into unnecessary neuroanatomy. Visualizing a full-size 6.5 × 50 screw on the screen projection prior to insertion of the PS allows the authors’ to ensure that the screw will avoid hitting any delicate surrounding anatomy. Essentially, the real-size virtual screw visualization and utilization of a small 3-mm match stick tip allow for minimal complications and certainty of the PS trajectory. A pedicle feeler is used to assess the integrity of the tract and confirm system accuracy. The next step uses an appropriate tap on the navigated power driver to access the vertebral body and finally to place and appropriate PS also using the navigated power driver.

Free-hand approach studies reported overall accuracies for pedicle screw placement as low as 71.9%; however, the range of accuracy for PS insertion using the free-hand technique has been reported as high as 91.3% [3,16,17,18,19,20,21,22]. The huge variability in accuracy can potentially be attributed to the learning curve required to master the free-hand technique, as the procedure is generally safe for experienced surgeons but results in complications for junior surgeons [6,9]. The variability can also be explained by the free-hand method’s reliance on surgeon technique preferences, not producing easily reproducible parameters for other surgeons [1]. In contrast to the free-hand method, studies illustrating the use of image-guided techniques have reported an accuracy range of 91.5% to 97.7%, overall much higher than the average accuracies reported for the free-hand technique [7,23,24,25,26,27]. With a surgical procedure that relies on high levels of precision to avoid proximity of the PS to vital structures and insertion of a large PS after removing anatomical features to access the proper region of PS placement, an image-guided approach may be more appropriate despite the limitations of cost, time, and exposure to radiation.

The results from the authors’ study support evidence in the literature that assisted techniques have higher accuracies of PS placement. With a 99.31% accuracy rate, the novel three-step virtual PS technique detailed in this paper illustrates consistency and precision. Image-guided neurosurgical techniques have been used for a while now, first used in cranial procedures and then by being slowly incorporated into the spinal axis [9]. Traditionally with image-guided spine surgery, pre-operative and intraoperative CT scans are utilized to visualize and align neuroanatomy with bony landmarks on a computer-generated image [11]. The result is a virtual guide that allows the surgeon to plan screw entry rather than relying on the removal of tissue to identify anatomy for free-hand screw placement.

The continued study and iteration of PS placement techniques is important because relative accuracies and revision of faulty screws determine patient outcomes. Screw revision is difficult, requires time and money, and results in patient complications [24]. With PS-based instrumentation remaining the best and strongest method for fixation in the thoracic, lumbar, and sacral spine, it is important to maximize precision and minimize complications. With spine surgery, PS placement still remains the most significant risk of patient morbidity [9]. With this risk, misplacement rates, and the variability of PS placement technique depending on institutional practices and surgeon preferences, there has been a recent push in the spine surgery field for usage of guided techniques [9]. The authors’ proposed three-step virtual PS technique implements such image-guided technology and successfully illustrates minimization of misplacement rates.

## 5. Conclusions

When implemented, the proposed navigated power driver with the virtual screw, three-step technique solves several problems associated with screw placement using only anatomical landmark observation. Without the need for extensive tissue dissection for entry point exposure and establishment of proper orientation, without the need for screw reposition due to intra- or post-operative complications, and by minimizing the steps and instrumentation required for PS entry, the authors have developed an accurate and safe method that augments existing techniques of navigated PS fixation. Low error rates of screw placement were seen with the novel three-step technique, resulting in an error rate of only 0.69% and a 0% morbidity. This technique shows promise to reduce the misplacement rate of screws, consequent revision rates, and associated surgical morbidities.

## Figures and Tables

**Table 1:** Patient demographics for virtual screw technique.

Number of Patients	23
Patient age range	19–65
Pathology treated	
Degenerative	15
Trauma	7
Neoplasm	1
Number of screws placed with virtual technique	
Thoracic	38
Lumbar	90
Sacral	16
Number of screws needing revision after Instrumentation, Intraoperative CT	1 (0.69%)
